# Pre-Operative nutrition In Neck of femur Trial (POINT) - carbohydrate loading in patients with fragility hip fracture: study protocol for a randomised controlled trial

**DOI:** 10.1186/1745-6215-15-475

**Published:** 2014-12-04

**Authors:** Iain K Moppett, Paul L Greenhaff, Ben J Ollivere, Theophillus Joachim, Dileep N Lobo, Martin Rowlands

**Affiliations:** Associate Professor and Honorary Consultant Anaesthetist, Anaesthesia and Critical Care Research Group, Division of Clinical Neuroscience, University of Nottingham, Nottingham, NG7 2UH UK; MRC/ARUK Centre for Musculoskeletal Ageing Research, ARUK Centre for Sport, Exercise and Osteoarthritis, School of Life Sciences, University of Nottingham, Nottingham, NG7 2UH UK; Consultant Orthopaedic and Trauma Surgeon Queen’s Medical Centre Campus, Nottingham University Hospitals NHS Trust, NG7 2UH Nottingham, UK; Associate specialist in Trauma and Orthopaedics, Lead for hip fracture management United Lincolnshire Hospitals, Pilgrim Hospital, Pilgrim Hospital, Sibsey Rd, Boston, Lincolnshire, PE21 9QS UK; Professor of Gastrointestinal Surgery Division of Surgery, University of Nottingham, Nottingham, NG7 2UH UK; Research Fellow, Anaesthesia and Critical Care Research Group, Division of Clinical Neuroscience, University of Nottingham, Nottingham, NG7 2UH UK

**Keywords:** insulin resistance/physiology, humans, carbohydrates/administration and dosage, preoperative care, hip fractures/surgery, energy metabolism/physiology, muscle, skeletal/metabolism

## Abstract

**Background:**

Trauma such as hip fracture initiates a neurohumoral stress response that changes the balance between anabolism and catabolism resulting in muscle breakdown and reduced mobilisation. Various studies have demonstrated a reduction in catabolism with pre-operative carbohydrate loading but only in an elective setting.

**Methods/Design:**

This is a two-centre, randomised double-blinded trial in the United Kingdom. Sample size will be 30 patients (approximately 15 from each centre).

Randomisation will be web based using computer-generated concealed tables. Both participants and investigators will be blinded to group allocation.

Participants will be >70 years of age, cognitively intact (Abbreviated Mental Score ≥7), able to give informed consent, and admitted directly through the emergency department with fractured neck of femur requiring hemiarthroplasty.

Intervention will consist of two carbohydrate drinks (Nutricia pre-Op) given the night before, and the morning of the surgery. The control will receive two placebo drinks of equal volume.

All participants will receive standard hospital care at the discretion of the clinical team. The primary outcome is the difference between groups in insulin resistance calculated by a glucose tolerance test administered pre-operatively and 24 hours postoperatively. Secondary endpoints will be changes in muscle carbohydrate metabolism (biopsy), mobility (Cumulative Ambulation Score) and subjective measures of tolerability.

**Discussion:**

This is a small-scale pilot study, investigating the benefits and tolerability of carbohydrate loading in an emergency setting in a frail elderly group with known high morbidity and mortality. Positive findings will provide the basis for a larger scale study.

**Trial registration:**

Current Controlled Trials ISRCTN91109766 (7 April 2014); NRES ref: 13/EM/0214

Trial Sponsor: University of Nottingham Ref.13036.

## Background

Trauma induces a widespread neurohumoral stress response. This results in changes in the balance between anabolic (growth) and catabolic (destructive) functions within the body. This has many effects, including increased catabolism, release of pro-inflammatory cytokines and activation of the sympatho-adrenal system.

Insulin resistance (IR) is believed to be a fundamental component regulating the change in the balance between anabolism and catabolism [1].

Insulin resistance is a metabolic state of reduced hepatic and peripheral (mainly muscle) responsiveness to insulin. Chronically, around 85% of Type II diabetics and 20% of non-diabetic individuals have biochemically defined insulin resistance [2]. In the peri-operative period, insulin resistance has a rapid onset [3], is predominantly of muscle origin and persists for a considerable period (weeks) following surgery. It appears to be, at least in part, a response to trauma and reduced energy intake [4].

The severity of insulin resistance is directly proportional to the magnitude of the surgical insult. It is also related to the development of postoperative complications [5]. Insulin resistance occurs in parallel with multiple other metabolic effects including decreased muscle carbohydrate oxidation, increased muscle catabolism, negative nitrogen balance, reduced muscle mass [6] and reduced muscle strength [7]. It is possible that they share a common signalling pathway.

There has been considerable work investigating the effect of differing forms of anaesthesia and surgery on the quality of this stress response. Minimally invasive surgery reduces, but does not eliminate, the stress response, and the beneficial effects may be dependent upon the type of surgery [8]. Regional anaesthesia, if complete, may abolish the adrenocortical, glycaemic and some of the inflammatory responses, but it does not abolish all of the cytokine responses to tissue trauma [9]. It has not been shown to improve outcome following hip fracture [10].

In recent years, a whole system approach to ameliorating the response to surgery has been introduced. The ‘Enhanced Recovery after Surgery’ approach (ERAS) aims to minimise all aspects of the surgical pathway that might impede recovery. Individual components of the bundle are of benefit with regard to surrogate and/or clinical outcomes. As a whole, the bundle has been associated with improved outcomes in a meta-analysis of randomised studies [11]. These studies, however, have only involved elective patients.

### Carbohydrate loading

The traditional approach to pre-operative preparation of patients involves a relatively prolonged period of pre-operative food and water starvation. This has been done to reduce the real, though small, risks of pulmonary aspiration of stomach contents. More recently it has been recognised that this approach induces the body’s starvation response. This may then be exacerbated by the catabolic effects of surgery. Several groups have demonstrated the beneficial effects of mitigating this by providing patients with carbohydrate drinks the evening before and morning of surgery.

Some of the beneficial effects seen in humans include reductions in insulin resistance and hyperglycaemia [12], postoperative nausea and vomiting [13], pre-operative discomfort [14] and preservation of muscle mass [15].

There is reasonable evidence for the safety of carbohydrate drinks [16]. Studies in volunteers [17], cardiac and general surgical [18] patients have not demonstrated increased gastric volumes when such drinks are given up to two hours before surgery.

Studies of oral carbohydrate loading have demonstrated a reduction in insulin resistance of approximately 50% in elective orthopaedic, colorectal and laparoscopic surgery [19], and the effect appears to be prolonged [8, 12, 16].

It is apparent that being in the fed versus the fasted state improves outcome following surgical stress; however, the exact reason for this is unclear. By avoiding depletion of liver glycogen reserves, there is a more rapid release of glucose from the liver into blood. Animal studies suggest that this results in a hyperosmolar state with possible benefits in fluid distribution and cardiovascular performance [19]. It may also affect immune function and reduce muscle protein catabolism, thereby maintaining muscle performance.

During the early phase of postoperative insulin resistance, the main defect is in the process of glucose uptake into the muscle. Specific glucose transporters (GLUT4) are not activated in the normal way by the action of circulating insulin [20].

More recent work has shown that the intracellular signalling systems that activate GLUT4, including PI-3-kinase, are downregulated while other signalling systems, which activate the inflammatory responses, are upregulated both in muscle [21] and fat tissue [22]. Animal work from the laboratory of one of the authors has demonstrated that a systemic inflammatory response increases the expression of pro-inflammatory cytokines in muscle, resulting in impaired PI-3-kinase signalling and the activation of downstream transcription FOXO factors. This has the effect of increasing the expression of muscle pyruvate dehydrogenase kinase 4 and muscle atrogene mRNA and protein. Collectively, these molecular responses impair muscle pyruvate dehydrogenase complex activation and carbohydrate use, as well as increasing proteasomal and cathepsin L activity and thereby muscle protein loss [23]. Translation of these findings has revealed that the same molecular events occur in intensive care patients [24] who are known to be insulin resistant. Furthermore, carbohydrate loading resulted in a reduction in the peri-operative induction of PDK4 mRNA, which inhibits oxidative carbohydrate metabolism [25].

These alterations in molecular signalling secondary to surgical stress are associated with a host of negative physiological effects postoperatively such as impaired glucose tolerance, decreased mitochondrial ATP production, increased gut permeability, increased free circulating fatty acids, decreased muscle glycogen levels and increased levels of blood lactate [26, 27].

Furthermore, it has been shown that increased muscle PDK4 expression inhibits the activity of pyruvate dehydrogenase complex (PDC), which controls the rate of muscle carbohydrate (CHO) oxidation in mitochondria. It is theorised that impaired PI3K/Akt1/FOXO signalling and the resultant upregulation of PDK4 are important factors underlying the development of muscle insulin resistance [28, 29].

### Muscle metabolism

Muscle metabolism and function is fundamental to postoperative recovery. Previous research on muscle metabolism following trauma and acute illness has largely focussed on organ-level changes in nitrogen balance and glucose metabolism. More recently research into the acute effects of illness on the transcriptional pathways involved in muscle homeostasis have shown that the changes are not a simple on/off phenomenon with some evidence of balancing anabolic processes being switched on shortly after the onset of sepsis. Although basal muscle metabolism is unaffected by age, the ability to incorporate amino acids following food may be different in the elderly [30] – so-called anabolic resistance.

Our study will investigate whether pre-operative CHO feeding has the ability to counter the cytokine mediated impairment of Akt phosphorylation caused by surgical stress, ultimately reducing the degree of insulin resistance.

### Hip fracture

Fragility hip fracture is a very significant burden to patients, carers and the health service [31]. Patients with fragility hip fracture have a poor outcome in terms of mortality and functional recovery [32, 33]. They also have a considerable hospital and rehabilitation length of stay. Early mobility is associated with better outcome [34], and the degree of mobility following discharge is linked to late mortality [35]. General muscle strength (not just lower limb strength) is associated with degree of functional recovery following hip fracture [36].

Careful multi-disciplinary pre-operative assessment and optimisation for surgery has been shown to improve functional outcomes and mortality. However, recent NICE guidance does not include the requirement for nutritional assessment. There is some evidence that nutritional intervention as part of a comprehensive hip fracture programme improves outcomes [37], and may reduce postoperative complications [38]. There are however no metabolic studies investigating pre-operative carbohydrate loading in this patient group.

There are few data regarding the stress response in the very elderly (the median age of hip fracture patients is approximately 84 years). Kudoh and colleagues [39] demonstrated differences between the very elderly and younger patients undergoing hip fracture surgery with regard to norepinephrine and cortisol responses, though the lack of analgesia in this study raises questions over relevance to modern practice. Pro-inflammatory cytokine responses appear to be augmented in the very elderly [40]. Sun and colleagues demonstrated a rise in plasma concentrations of pro-inflammatory cytokines following admission with hip fracture in the elderly, and the magnitude of this response was predictive of 6 month and 12 month mortality [41]. Studies of insulin resistance have had few or no patients over 80 [42, 43].

Qualitatively, the hormonal response following minor surgery appears similar in younger and in older (70 to 75 years) patients [44, 45]. Previous researchers have demonstrated an early and persisting loss of muscle mass following hip fracture [46], which may be one reason for the decline in function and persistent insulin resistance seen following injury. These data would suggest that if practical strategies to reduce insulin resistance were available, there may be possible outcome benefits for patients following hip fracture.

Previous studies using pharmacological modulation of muscle metabolism in this patient group have shown conflicting results. Studies using human growth hormone or similar drugs have reported increased side effects in the treatment groups [47, 48].

Postoperative nutritional supplementation has had some favourable results [49] but has suffered with poor compliance. A Cochrane review of nutritional supplementation found only weak evidence of benefit [50]. One reason for this may be that the intervention is insufficient in patients who are already in a catabolic state after surgery. Pre-surgical intervention may not only provide nutrition but also modulate the catabolic response.

There has been a relatively successful national drive to reduce the time between admission and surgery for patients with hip fracture. According to the latest results from the National Hip Fracture Database 2013 71.4% of patients received their operation within 36 hours [51]. Nonetheless, this still provides a considerable period when patients may be kept nil by mouth awaiting operation. Accurate data for true starvation periods are scarce. A small audit from a UK District General Hospital found ‘average’ starvation times after 7 am of 4 to 6 hours, which should be added to the 7 hours from midnight [52]. Although most patients are now given intravenous rehydration whilst waiting for theatre, food is less commonly given and nausea is a common complication of the injury, pain and the opioids given for analgesia.

There is a small amount of evidence of impaired glucose homeostasis in hip fracture patients. Liu found evidence of postoperative hyperglycaemia in around 70% of patients [41]. Around 40% of patients from the recently completed NOTTS study [53, 54] are hyperglycaemic prior to induction of anaesthesia.

However, the evidence from elective surgery may not be directly applicable to hip fracture patients. First, by definition, these patients have already suffered one traumatic insult (the injury) so the catabolic processes may have already begun. Second, the carbohydrate loading studies have generally used an 800-ml drink the night before surgery and a 200-ml drink on the morning of surgery. Although this is well tolerated in elective surgical patients, it may not be so well tolerated by frail elderly patients. Third, approximately 50% of hip fracture surgery is performed under spinal anaesthesia [51]. There is some evidence from elective surgery that this may diminish the impact of carbohydrate loading, presumably due to the reduction in the stress response.

## Methods/Design

### Study objectives

#### Primary aims

To investigate whether pre-operative carbohydrate loading (using Nutricia pre-op drinks) results in an improvement in insulin resistance in patients undergoing hemiarthroplasty for a fragility fracture of the neck of femur.

#### Secondary aims

Does pre-operative carbohydrate loading in elderly hip fracture patients have other benefits in the post-operative period? The following will be evaluated to determine this:

1. Muscle metabolism.
2. Functional mobility (measure with the Cumulative Ambulation Score).
3. Infectious/cardiovascular complications.
4. Length of acute hospital stay.

### Study design

This is a prospective, parallel group, double-blind (participant and investigators), randomised controlled clinical trial.

Two centres will participate: Queen’s Medical Centre campus of Nottingham University Hospitals and Pilgrim Hospital, United Lincolnshire Hospitals, Boston.

Recruitment started in September 2014, and total recruitment in expected to take 12 to 18 months.

### Randomisation and blinding

Randomisation (on a one-to-one basis) will be provided by a password-protected web-based randomisation service, with sequence not revealed until data lock. Participants will be randomised after x-ray confirmation of a displaced intracapsular fracture of the neck of femur and appropriate assessment and counselling from a member of the research team.

The investigators, patient, nurses and data-collection staff will all be blinded to treatment allocation. Drinks will be prepared by a separate member of staff who will not be blinded for practical reasons but who will not take part in any aspect of the study apart from preparation of the drinks.

The assessment of Cumulative Ambulation Score will be made by the ward physiotherapists treating the patient. The multiprofessional team makes the decision that a patient is medically fit for discharge when all are satisfied that the participant has no ongoing needs for acute hospital care. This team is blinded to participant allocation.

### Selection and withdrawal of participants

#### Recruitment

Participants will be identified by nurses in the Emergency Department, who will then inform a member of the research team about the presence of the patient. Once a patient has been identified, a member of the research team will approach the patient and conduct the initial screening and consenting of the patient. Active participation in the study will be until the second glucose tolerance test has been performed 24 hours after surgical fixation.

Participants will be informed that participation is voluntary and that they are free to withdraw at any time without affecting their care.

Data on time to discharge and mortality are routinely collected on all hip fracture patients at each institution, and participation in the trial will involve consent for this data to be used.

Patients unable to provide informed consent will be excluded from the trial. Patients for whom language will be a barrier will also be excluded due to the small scale nature of the study. Patient information will not be available in languages other than English. In practice, non-English speakers represent a very small number of patients in this population group.

### Inclusion criteria

Inclusion criteria include the following:

1. Primary hip fracture listed for surgical repair using hemiarthroplasty.
2. Age over 70 years.
3. Able to give informed consent.

### Exclusion criteria

Exclusion criteria include the following:

1. Pre-existing diabetes (type I or II).
2. Intercurrent infection.
3. Ongoing participation in another interventional clinical trial.
4. Previous intolerance to carbohydrate drinks.
5. Any condition or impairment, which, in the view of the investigator, would prohibit the patient from full participation in the study.

## Informed consent

Informed consent is obtained from every participant. The capacity for providing consent is assessed routinely by the orthopaedic team, which decides whether the patient is competent to provide consent for the surgical procedure. If the orthopaedic team deems the patient unable to consent to surgery, then the patient will be deemed incapable of consenting to enter the study. A member of the research team also performs an additional check of the participant’s ability to provide consent immediately prior to starting the study. All members of the research team are trained at obtaining consent in accordance with guidance for good clinical practice [55].

The trial received favourable ethical approval from the NRES Committee East Midlands-Nottingham 1 on 15 July 2013.

### Study intervention

All patients enrolled into the trial will receive a baseline glucose tolerance test a minimum of four hours after their last oral intake. Routine practice at both study centres is to place patients with diagnosed hip fractures nil by mouth until an operating theatre time is identified. Once this test is performed patients allocated to the active group will receive 400 ml of carbohydrate drink (Nutricia Pre-op). Patients in the control arm will receive 400 ml of diet (sugar free) lemon drink. This will be administered by the investigator from an opaque jug to reduce identification of the drink. A further 400 ml of either carbohydrate drink or of placebo will be administered by nursing staff at 06:00 hours on the morning of surgery. This is to allow a minimum of two hours fasting pre-operatively, which is standard policy at both sites.

Three muscle biopsies will be performed in total, taken from the *vastus lateralis*: one after anaesthesia but before surgery in the non-fractured leg, with sample two being taken after wound closure but before wake up (for general anaesthesia) or resolution of regional anaesthesia. The third muscle biopsy will be taken 24 hours after the end of surgery at approximately the same time as the second glucose tolerance test. All muscle biopsies will be snap frozen in liquid nitrogen and stored at -80°C before transport to the muscle physiology laboratory for analysis.

### Standard care

Standard care will be identical in both groups. Only the administration of the carbohydrate drink will differ between groups.

All patients are admitted to dedicated trauma wards and cared for in accordance with UK ‘Best Practice Tariff’ [56, 57].

This includes assessment by orthogeriatricians, operation within 36 hours of admission, and assessment of bone health and falls. All patients are cared for under a hip fracture care pathway, which involves rapid assessment and admission from the emergency department, intravenous crystalloid infusions from the time of admission, and multiprofessional care and discharge planning. Operations are performed in dedicated trauma theatres by appropriately experienced surgeons and anaesthetists.

The surgical stress response *per se* generates insulin resistance; therefore the protocol does not allow a *de novo* diagnosis of diabetes. However, glucose tolerance test results will be available to the clinical team to make appropriate clinical decisions.

### Concomitant medication

Analgesia will be provided in accordance with normal practice at the study centre. Non-steroidal anti-inflammatory drugs are not routinely prescribed for this group of patient due to their side effects. Other medications will be prescribed by the attending medical staff as appropriate for each individual.

### Concomitant treatments

Anaesthesia will be at the discretion of the attending anaesthetist. Other post-operative therapies such as physiotherapy will be in accordance with routine hospital practice at the relevant centre.

### Laboratory analysis

Glucose tolerance test blood will be analysed on a bedside blood glucose analyser (Hemocue, Brea CA, USA). C-peptide and insulin levels, along with all muscle biopsy samples, will be analysed by Professor Greenhaff’s team at the MRC-ARUK Centre for Musculoskeletal Ageing Research, University of Nottingham.

### Muscle metabolism

Laboratory analysis will be performed by Professor Greenhaff’s team at the School of Biomedical Sciences University of Nottingham. This will include PCR/western blot and spectrophotometric analysis. The following parameters will be examined:

1. TNF / IL6 mRNA expression, extent of activation of inflammatory processes, assessed using real-time PCR.
2. AKT protein phosphorylation-phosphorylates FOXO, leading to decreased FOXO activity, assessed using western blotting.
3. FOXO 1 and 3 protein phosphorylation, induces PDK4 transcription, assessed using western blotting.
4. PDK 2 and PDK4 (pyruvate dehydrogenase lipoamide kinase isozyme 4) mRNA and protein expression, muscle-specific isoform known to inhibit PDC activation, assessed using real-time PCR and western blotting.
5. Glycogen, muscle store of carbohydrate, assessed using spectrophotometry.
6. PDC (pyruvate dehydrogenase complex) protein activation status, rate limiting enzyme for mitochondrial carbohydrate oxidation, assessed using a radioimmunoassay.

## Discussion

This is a small-scale pilot study. Preoperative carbohydrate loading has been shown to be effective in suppressing the insulin resistance that develops in other postsurgical populations, but its use has never been investigated in an emergency hip fracture population.

Carbohydrate loading is a relatively cheap intervention with little or no side effects which, if shown to be of benefit, could be rolled out across the whole patient population and included as part of an emergency hip fracture enhanced recovery programme. Conversely, it may be of little benefit and resources could be directed elsewhere. The secondary data on the metabolic effects of trauma in the elderly will provide important insights into ageing physiology that will be of increasing importance as the population demographic changes.

It is hoped that if this study shows positive effects, it will serve as a guide for larger studies investigating carbohydrate loading in emergency patients.

### Trial status

NUH NHS R and I approval was gained on 24 April 2014

The trial started recruiting September 2014.

## Abbreviations

AKT: protein kinase
FOXO: Forkhead box protein
IL-6: Interleukin 6
IR: insulin resistance
mRNA: messenger RNA
PCR: polymerase chain reaction
PDC: pyruvate dehydrogenase complex
PDK2/4: pyruvate dehydrogenase lipoamide kinase isozyme 2/4
TNF: tumour necrosis factor.

## References

[1] Thorell ANJ, Ljungqvist O (1999). Insulin resistance: a marker of surgical stress. Curr Opin Clin Nutr Metab Care.

[2] Beck Nielsen H (1999). General characteristics of the insulin resistance syndrome: prevalence and heritability. European group for the study of insulin resistance (EGIR). Drugs.

[3] Ljungqvist O (2010). Insulin resistance and outcomes in surgery. J Clin Endocrinol Metab.

[4] Nygren J, Thorell A, Brismar K, Karpe F, Ljungqvist O (1997). Short-term hypocaloric nutrition but not bed rest decrease insulin sensitivity and IGF-I bioavailability in healthy subjects: the importance of glucagon. Nutrition.

[5] Sato H, Carvalho G, Sato T, Lattermann R, Matsukawa T, Schricker T (2010). The association of preoperative glycemic control, intraoperative insulin sensitivity, and outcomes after cardiac surgery. J Clin Endocrinol Metab.

[6] Hedström M, Sääf M, Dalén N (1999). Low IGF-I levels in hip fracture patients. A comparison of 20 coxarthrotic and 23 hip fracture patients. Acta Orthop Scand.

[7] Ljungqvist O, Soop M, Hedstrom M (2007). Why metabolism matters in elective orthopedic surgery: a review. Acta Orthop.

[8] Veenhof AAFA, Sietses C, Von Blomberg BME, Van Hoogstraten IMW, VD Pas MHGM, Meijerink WJHJ, VD Peet DL, VD Tol MP, Bonjer HJ, Cuesta MA (2011). The surgical stress response and postoperative immune function after laparoscopic or conventional total mesorectal excision in rectal cancer: a randomized trial. Int J Color Dis.

[9] Ahlers O, Nachtigall I, Lenze J, Goldmann A, Schulte E, Hohne C, Fritz G, Keh D (2008). Intraoperative thoracic epidural anaesthesia attenuates stress-induced immunosuppression in patients undergoing major abdominal surgery. Br J Anaesth.

[10] White SM, Griffiths R, Moppett IK (2012). Type of anaesthesia for hip fracture surgery - the problems of trial design. Anaesthesia.

[11] Varadhan KK, Neal KR, Dejong CH, Fearon KC, Ljungqvist O, Lobo DN (2010). The enhanced recovery after surgery (ERAS) pathway for patients undergoing major elective open colorectal surgery: a meta-analysis of randomized controlled trials. Clin Nutr.

[12] Soop M, Nygren J, Myrenfors P, Thorell A, Ljungqvist O (2001). Preoperative oral carbohydrate treatment attenuates immediate postoperative insulin resistance. Am J Physiol Endocrinol Metab.

[13] Hausel J, Nygren J, Thorell A, Lagerkranser M, Ljungqvist O (2005). Randomized clinical trial of the effects of oral preoperative carbohydrates on postoperative nausea and vomiting after laparoscopic cholecystectomy. Br J Surg.

[14] Hausel J, Nygren J, Lagerkranser M, Hellstrom PM, Hammarqvist F, Almstrom C, Lindh A, Thorell A, Ljungqvist O (2001). A carbohydrate-rich drink reduces preoperative discomfort in elective surgery patients. Anesth Analg.

[15] Yuill KA, Richardson RA, Davidson HI, Garden OJ, Parks RW (2005). The administration of an oral carbohydrate-containing fluid prior to major elective upper-gastrointestinal surgery preserves skeletal muscle mass postoperatively–a randomised clinical trial. Clin Nutr.

[16] Ljungqvist O, Nygren J, Thorell A (2002). Modulation of post-operative insulin resistance by pre-operative carbohydrate loading. Proc Nutrition Society.

[17] Lobo DN, Hendry PO, Rodrigues G, Marciani L, Totman JJ, Wright JW, Preston T, Gowland P, Spiller RC, Fearon KC (2009). Gastric emptying of three liquid oral preoperative metabolic preconditioning regimens measured by magnetic resonance imaging in healthy adult volunteers: a randomised double-blind, crossover study. Clin Nutr.

[18] Yagci G, Can MF, Ozturk E, Dag B, Ozgurtas T, Cosar A, Tufan T (2008). Effects of preoperative carbohydrate loading on glucose metabolism and gastric contents in patients undergoing moderate surgery: a randomized, controlled trial. Nutrition.

[19] Ljungqvist O, Boija PO, Esahili H, Larsson M, Ware J (1990). Food deprivation alters liver glycogen metabolism and endocrine responses to hemorrhage. Am J Physiol.

[20] Awad S, Constantin-Teodosiu D, Macdonald IA, Lobo DN (2009). Short-term starvation and mitochondrial dysfunction - a possible mechanism leading to postoperative insulin resistance. Clin Nutr.

[21] Witasp A, Nordfors L, Schalling M, Nygren J, Ljungqvist O, Thorell A (2009). Increased expression of inflammatory pathway genes in skeletal muscle during surgery. Clin Nutr.

[22] Witasp A, Nordfors L, Schalling M, Nygren J, Ljungqvist O, Thorell A (2010). Expression of inflammatory and insulin signaling genes in adipose tissue in response to elective surgery. J Clin Endocrinol Metab.

[23] Crossland H, Constantin-Teodosiu D, Gardiner SM, Constantin D, Greenhaff PL (2008). A potential role for Akt/FOXO signalling in both protein loss and the impairment of muscle carbohydrate oxidation during sepsis in rodent skeletal muscle. J Physiol.

[24] Constantin D, McCullough J, Mahajan RP, Greenhaff PL (2011). Novel events in the molecular regulation of muscle mass in critically ill patients. J Physiol.

[25] Awad S, Constantin-Teodosiu D, Constantin D, Rowlands BJ, Fearon KC, Macdonald IA, Lobo DN (2010). Cellular mechanisms underlying the protective effects of preoperative feeding: a randomized study investigating muscle and liver glycogen content, mitochondrial function, gene and protein expression. Ann Surg.

[26] Varadhan KK, Atkins RP, Constantin-Teodosiu D, Elaine B, Perkins AC, Greenhaff PL, Lobo DN (2011). Gastrointestinal surgery mediated increases in gut permeability and expression of IL6 and PDK4 mRNAs in quadriceps muscle may underpin the post-operative increase in whole-body insulin resistance in humans. J Am Coll Surg.

[27] Atkins RP, Varadhan KK, Constantin-Teodosiu D, Lobo DN, Greenhaff PL (2011). Rates of skeletal muscle mitochondrial ATP production are reduced during elective abdominal surgery in humans. J Am Coll Surg.

[28] Murton AJ, Constantin D, Greenhaff PL (2008). The involvement of the ubiquitin proteasome system in human skeletal muscle remodelling and atrophy. Biochim Biophys Acta.

[29] Kim YI, Lee FN, Choi WS, Lee S, Youn JH (2006). Insulin regulation of skeletal muscle PDK4 mRNA expression is impaired in acute insulin-resistant states. Diabetes.

[30] Koopman R, Van Loon LJ (2009). Aging, exercise, and muscle protein metabolism. J Appl Physiol (1985).

[31] White SM, Griffiths R (2011). Projected incidence of proximal femoral fracture in England: a report from the NHS Hip Fracture Anaesthesia Network (HIPFAN). Injury.

[32] Maxwell MJ, Moran CG, Moppett IK (2008). Development and validation of a preoperative scoring system to predict 30 day mortality in patients undergoing hip fracture surgery. Br J Anaesth.

[33] Wiles MD, Moran CG, Sahota O, Moppett IK (2011). Nottingham Hip fracture score as a predictor of one year mortality in patients undergoing surgical repair of fractured neck of femur. Br J Anaesth.

[34] Foss NB, Kristensen MT, Kehlet H (2006). Prediction of postoperative morbidity, mortality and rehabilitation in hip fracture patients: the cumulated ambulation score. Clin Rehabil.

[35] Fox KM, Hawkes WG, Hebel JR, Felsenthal G, Clark M, Zimmerman SI, Kenzora JE, Magaziner J (1998). Mobility after hip fracture predicts health outcomes. J Am Geriatr Soc.

[36] Beloosesky Y, Weiss A, Manasian M, Salai M (2010). Handgrip strength of the elderly after hip fracture repair correlates with functional outcome. Disabil Rehabil.

[37] Pedersen SJ, Borgbjerg FM, Schousboe B, Pedersen BD, Jorgensen HL, Duus BR, Lauritzen JB (2008). A comprehensive hip fracture program reduces complication rates and mortality. J Am Geriatr Soc.

[38] Gunnarsson AK, Lonn K, Gunningberg L (2009). Does nutritional intervention for patients with hip fractures reduce postoperative complications and improve rehabilitation?. J Clin Nurs.

[39] Kudoh A, Ishihara H, Matsuki A (1999). Response to surgical stress in elderly patients and Alzheimer’s disease. Can J Anesth.

[40] Kudoh A, Katagai H, Takazawa T, Matsuki A (2001). Plasma proinflammatory cytokine response to surgical stress in elderly patients. Cytokine.

[41] Sun T, Wang X, Liu Z, Chen X, Zhang J (2011). Plasma concentrations of pro- and anti-inflammatory cytokines and outcome prediction in elderly hip fracture patients. Injury.

[42] Mathur S, Plank L, McCall J, Shapkov P, McIlroy K, Gillanders L, Merrie A, Torrie J, Pugh F, Koea J (2010). Randomized controlled trial of preoperative oral carbohydrate treatment in major abdominal surgery. Br J Surg.

[43] Perrone F, Da Silva Filho AC, Adorno IF, Anabuki NT, Leal FS, Colombo TDA, Silva BD, Dock Nascimento DB, Damiao A, De Aguilar-Nascimento JE (2011). Effects of preoperative feeding with a whey protein plus carbohydrate drink on the acute phase response and insulin resistance. A randomized trial. Nutr J.

[44] Blichert-Toft M, Christensen V, Engquist A, Fog-Moller F, Kehlet H, Madsen SN, Skovsted L, Thode J, Olgaard K (1979). Influence of age on the endocrine-metabolic response to surgery. Ann Surg.

[45] Oyama T, Taniguchi K, Takazawa T, Matsuki A, Kudo M (1980). Effect of anaesthesia and surgery on endocrine function in elderly patients. Can Anaesth Soc J.

[46] Fox KMMJ, Hawkes WG, Yu Yahiro J, Hebel JR, Zimmerman SI, Holder L, Michael R (2000). Loss of bone density and lean body mass after hip fracture. Osteoporos Int.

[47] Adunsky A, Chandler J, Heyden N, Lutkiewicz J, Scott BB, Berd Y, Liu N, Papanicolaou DA (2011). MK-0677 (ibutamoren mesylate) for the treatment of patients recovering from hip fracture: a multicenter, randomized, placebo-controlled phase IIb study. Arch Gerontol Geriatr.

[48] Yeo AL, Levy D, Martin FC, Sonksen P, Sturgess I, Wheeler MM, Young A (2003). Frailty and the biochemical effects of recombinant human growth hormone in women after surgery for hip fracture. Growth Hormon IGF Res.

[49] Lawson RM, Doshi MK, Barton JR, Cobden I (2003). The effect of unselected post-operative nutritional supplementation on nutritional status and clinical outcome of orthopaedic patients. Clin Nutr.

[50] Avenell A, Handoll HH (2010). Nutritional supplementation for hip fracture aftercare in older people. Cochrane Database Syst Rev.

[51] **The National Hip Fracure Database National Report** 2013. http://www.nhfd.co.uk/20/hipfractureR.nsf/4e9601565a8ebbaa802579ea0035b25d/566c6709d04a865780257bdb00591cda/$FILE/onlineNHFDreport.pdf

[52] Moir J, Dixon P (2011). Time to surgery and starvation times of patients with neck of femur fractures: a prospective audit. Online J of Clin Audits.

[53] Wiles MD, Whiteley WJ, Moran CG, Moppett IK (2011). The use of LiDCO based fluid management in patients undergoing hip fracture surgery under spinal anaesthesia: neck of femur optimisation therapy - targeted stroke volume (NOTTS): study protocol for a randomized controlled trial. Trials.

[54] Moppett IK, Rowlands M, Mannings A, Moran CG, Wiles MD (2014). The use of LiDCO based fluid management in patients undergoing hip fracture surgery under spinal anaesthesia (NOTTS): a randomised clinical trial and updated systematic review. Br J Anaesth.

[55] International Conference on Harmonisation of Technical Requirements for Registration of Pharmaceuticals for Human Use (ICH) (1996). Guidance for Industry E6 Good Clinical Practice.

[56] **British Orthopaedic Association Standards for Trauma (BOAST): Boast 1 Version 2: Hip Fracture in the Older Person** 2012. http://www.boa.ac.uk/LIB/LIBPUB/Documents/BOAST%201%20Version%202%20-%20Hip%20Fracture%20in%20the%20Older%20Person%20-%202012.pdf

[57] Department of Health Payment by Results Team (2013). Payment by Results Guidance for 2013–14.

